# Ventrolateral but not Dorsolateral Prefrontal Cortex tDCS effectively impact emotion reappraisal – effects on Emotional Experience and Interbeat Interval

**DOI:** 10.1038/s41598-018-33711-5

**Published:** 2018-10-17

**Authors:** Lucas M. Marques, Letícia Y. N. Morello, Paulo S. Boggio

**Affiliations:** Social and Cognitive Neuroscience Laboratory and Developmental Disorders Program, Center for Health and Biological Sciences, Mackenzie Presbyterian University, 01241-001 Sao Paulo, Brazil

## Abstract

Emotions can be understood as behavioral, physiological, and subjective individual’s alteration due to a given situation. Several times, an efficient regulation of these emotions can promote psychological and social survival. It has been demonstrated that the Prefrontal Cortex (PFC) presents a relevant role in cognitive control, especially during emotion regulation strategies. However, evidence for the role of the PFC and emotional regulation comes mostly from neuroimaging experiments lacking from causal information. Transcranial Direct Current Stimulation (tDCS) has been shown to be an efficient noninvasive neuromodulation technique capable to address causal hypothesis. The aim of this study was to investigate the role of two regions of the PFC (Dorsolateral and Ventrolateral region) on different strategies of emotional reappraisal during the observation of negative images. 180 undergraduate students (mean age 21,75 ± 3,38) participated in this study, divided in two experiments (Dorsolateral PFC - n = 90; Ventrolateral PFC - n = 90). As not expected, DLPFC tDCS did not modulate the responses on the emotional regulation task. However, VLPFC tDCS resulted in less negative valence of negative images as well as decreased cardiac interbeat interval on earlier moments of emotional processing. These findings supports the general view about the role of the PFC on emotional regulation and, at the same time, advances the field by providing evidence that evaluation of negative stimuli is much more based on the VLPFC than on the DLPCF.

## Introduction

Among its most diverse definitions, emotion can be understood as a physiological alteration and/or a cognitive process which drives the individual towards an action important for one’s survival^1^. Typically, efficient emotional regulation allows for healthy adaptation to one’s social and emotional environment^2^. As such, multiple strategies for emotional regulation have been investigated and developed, such as cognitive reappraisal^3^, which is characterised by a process of reappraisal of the cognitive label given to a specific stimulus, stimulating the exacerbation/approach (up-regulation) or attenuation/avoidance (down-regulation) of an emotional effect.

In parallel, with the development of novel methods in neuroscience, we have gained a greater understanding about the function of cortical and subcortical structures during emotional regulation^4^. Beyond the involvement of cerebral structures such as the insular cortex and the amygdala^5^, the cognitive reappraisal of emotional stimuli recruits cortical structures such as the anterior cingulate cortex (ACC), dorsolateral prefrontal cortex (DLPFC) and the ventrolateral prefrontal cortex (VLPFC) (for a review, see Kalisch^6^ and Buhle *et al*.^7^). Even so, there is much to be learned about the specific role played by each cortical region in cognitive reappraisal; a research topic that might advance by using neuromodulation.

Among the main techniques of neuromodulation, Transcranial Direct Current Stimulation (tDCS) has been shown to be an effective non-invasive technique for research conducted with both healthy^8^ and clinical subjects^9^ in the domain of social cognition^10^. The technique is based on the application of a continuous, low-intensity electrical current, where one typically observes patterns of excitatory/inhibitory modulation in the cortical regions immediately below the positioned electrodes, depending on the polarity used^11^.

Until recently, few studies have sought to understand the effect of tDCS during emotional regulation. One of these is a recent study by^12^, which showed that anodal tDCS to the right DLPFC resulted in a significant increase in subjects’ capacity for cognitive reappraisal as compared to a sham condition, both in terms of the up-regulation as well as the down-regulation of the current emotion, indicating a distinct role of the DLPFC in the cognitive control of emotion. However, this study applied anodal stimulation to the right hemisphere only and did not employ an experimental design which allowed for the simultaneous investigation of the function of both hemispheres in this cortical region. Concomitantly, a more recent study by^13^ showed that anodal tDCS to the right DLPFC but not the left, is correlated with a greater cognitive control during emotional regulation, mainly with negative emotional images. These findings are consistent with another recent study by^14^, in which the authors showed an effective role of the right DLPFC in the control of the emotional impact from negatively valent videos about pain. It is important to emphasize that these tDCS findings are also in line with TMS findings (for a review, see Lantrip *et al*.^15^), reinforcing the importance of these PFC regions in the emotional control.

In parallel, other studies have sought to understand the role of the VLPFC in emotional regulation. A study by^16^ showed that anodal tDCS to the right VLPFC during a social ostracism task can reduce subjects’ self-reported feelings of discomfort and pain, when compared to the results from the sham group. More recently, the same group showed that subjects receiving anodal tDCS to the right VLPFC in the same experimental paradigm reported lower levels of aggression^17^. Taken together, these studies suggest that the DLPFC and VLPFC exert a similar effect on processes of emotional control, albeit in different ways. Nevertheless, it is important to monitor the effect of the simultaneous neuromodulation of both cerebral hemispheres and of both regions during the performance of tasks of emotional control.

As such, the current study sought to investigate the role of the prefrontal cortex in different strategies of cognitive reappraisal, specifically the role of inter-hemispheric modulation of the DLPFC and VLPFC. To this end, two experiments were conducted using tDCS during cognitive reappraisal by healthy subjects, with Experiment 1 investigating the DLPFC and Experiment 2 investigating the VLPFC.

## Experiment 1 – DLPFC tDCS

### Materials and Methods

#### Participants

90 university students participated in the experiment. The criteria for inclusion were: (i) no reported neurological, psychiatric or severe psychological impairments; (ii) right-handedness; (iii) aged between 18 and 35; and (iv) no use of medication affecting the central nervous system. A between-subjects design was employed, controlling for possible practice effects in the task of cognitive reappraisal. The study was approved by the Institutional Review Board of the Mackenzie Presbyterian University and by the National Ethics committee (SISNEP, Brazil), all participants provided written informed consent, and all experiments were performed in accordance with relevant guidelines and regulations.

#### Experimental Procedure

The participants were initially tested on the Edinburgh Handedness Inventory^18^, the Emotion Regulation Questionnaire (ERQ), with both subcomponents, the ERQ-CR and ERQ-S (Cognitive Reappraisal and Suppression, respectively^19^), and both Beck inventories (the BAI and BDI^20^). Furthermore, participants also completed the Positive and Negative Affect Scale (PANAS^21^). After the completion of all questionnaires, the sites for electrode positions were cleaned with ethanol solution, followed by the placement of electrocardiographic electrodes (ECG) electrodes. After this stage tDCS montage was initiated, with the corresponding steps of measurement, positioning and fade-in. After that, participants were briefed on the experimental task.

Participants remained comfortably seated during the experiment at approximately 1 metre from the monitor. After the task was completed and tDCS fade-out ended, the electrodes were removed from the participant and cleaned. At this stage, the participants completed the PANAS scale a second time.

#### Cognitive Reappraisal Task

The cognitive reappraisal task used in the experiment was adapted from^2^, characterized by the observation and evaluation of images of high emotional arousal and negative valence. Subjects performed the evaluation in a two-step process through the input of a numerical key for: (i) valence estimation and (ii) arousal estimation. The experimental sequence was as follows: initially, instructions about which emotional strategy was to be adopted (i.e. up- or down-regulation), were presented for 1000 ms; followed by a target image presented for 10000 ms; followed by an evaluation of the image’s emotional valence with no time limit for the subject’s response, and evaluation of the image’s emotional arousal with no time limit too; and finally by a screen to prepare subjects for the next trial, presented for 1000 ms. All images were presented using E-prime 2.0® (Psychological Software Inc.) and a 32-inch widescreen monitor (Samsung 320BX®).

The three cognitive reappraisal strategies which the participants were instructed to use were: (i) to increase the current emotion (“look at the situation in the image and imagine the situation as worse than presented”); (ii) to decrease the current emotion (“look at the situation in the image and imagine the situation as better than presented”) and (iii) to maintain the current emotion (“look at the situation in the image passively”). The strategies were presented in an equal number and the order of presentation was randomly determined. The Self-Assessment Manikin (Sam^22^) was used as a method of evaluation with two 9 points Likert scales to evaluate: (i) arousal level (from not intense at all to extremely intense); and (ii) valence (from extremely negative to extremely positive). Before the experimental task began, subjects completed a training session in which they practised using each cognitive reappraisal strategy. In all, 72 images with negative emotional content were used (24 images for each strategy). All target images had low levels of emotional valence and high levels of emotional arousal (see Appendix), considering the normative values for the city of São Paulo^23^ for images from the International Affective Picture System (IAPS^24^). The practice session and the experimental task lasted approximately 40 minutes.

#### Transcranial Direct Current Stimulation (tDCS)

**Figure 1:**
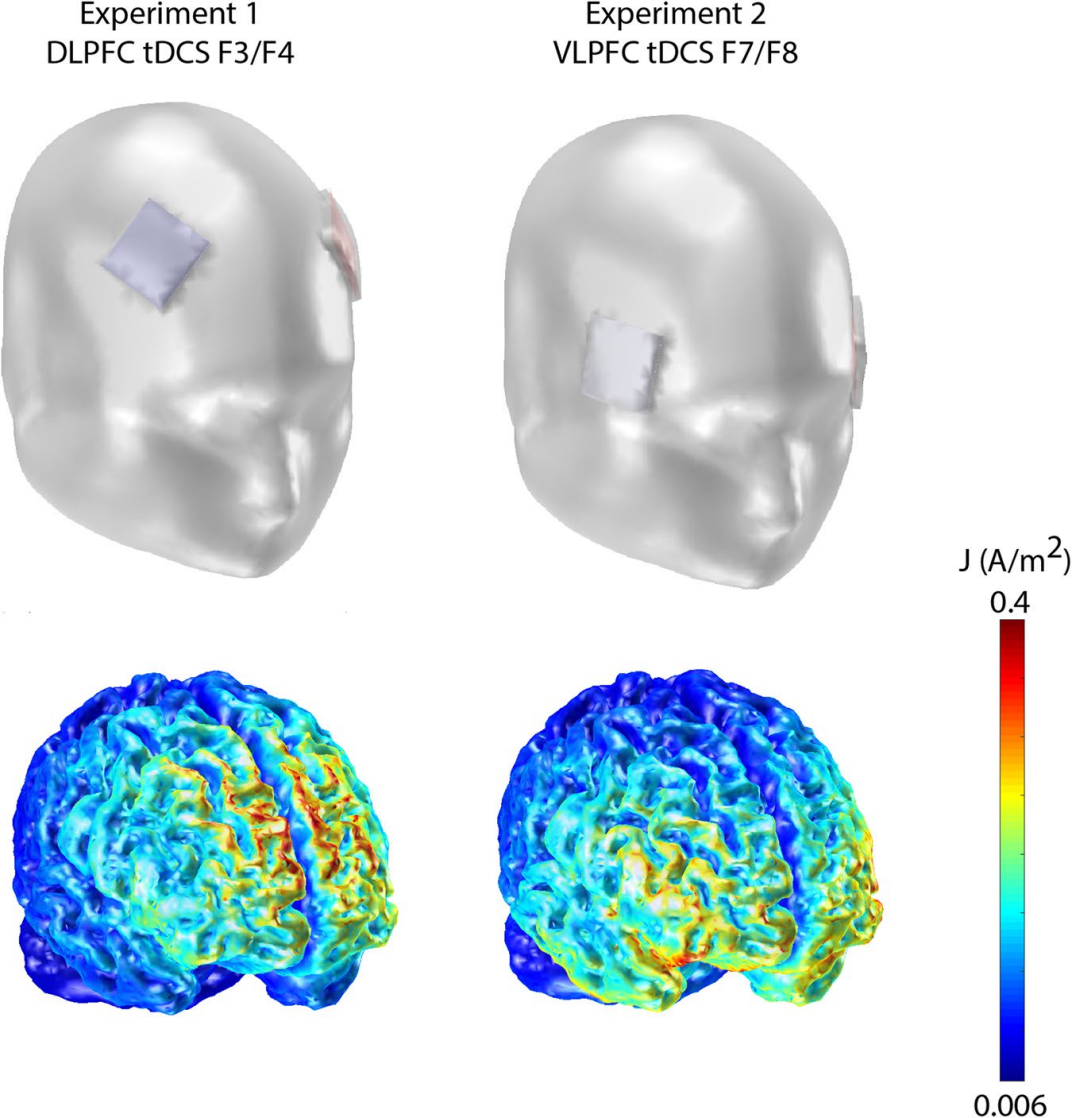
tDCS montages for Experiments 1 and 2.

The equipment used in the experiment included a low-intensity DC stimulator; two rubber electrodes, each with an individual surface area of 16 cm^2^, covered by sponges soaked in a saline solution and placed over the target area; as well as elastic strips to hold the electrodes in their specified place^8^. The system of electrode positioning (Fig. 1) followed the classic 10–20 system of EEG electrodes placement by Jasper^25^, used in studies by, Feeser *et al*.^12^ and Riva *et al*.^17^, with three separate tDCS montage conditions with each subject tested on only one condition: (1) anode positioned on F3 and cathode positioned on F4 (ANF3/CATF4); (2) anode positioned on F4 and cathode positioned on F3 (ANF4/CATF3); (3) sham stimulation, with the same montage as condition ANF3/CATF4. Furthermore, considering that tDCS is not particularly focal and to reduce this limitation, we used a MATLAB toolbox called COMETS2^26^. This toolbox provides current flow and current density calculated based on a realistic human head model composed of scalp, skull, CSF, and brain. Figure 1 shows a simulation of the electric field distribution on both Experiments.

Both the sham stimulation and the experimental stimulation were conducted at a current of 1.5 mA (0.094 mA/cm^2^ current density), with a fade-in and fade-out of 15 seconds each. The duration of stimulation was of 20 minutes for the experimental stimulation, including 5 minutes of stimulation conducted prior to the start of the task, and 30 seconds only in the sham condition. The participants were blinded for the stimulation condition.

#### Interbeat Interval measure (IBI)

Considering psychophysiological measures of emotional response, the studies typically use SCR measures Electrodermal Activity technique and the Skin Conductance Analysis (as tested by Feeser *et al*.^12^). Here we chose to use the Electrocardiography, since some studies demonstrated significant impact of tDCS on cardiac response^27–29^. Furthermore, some findings emphasize the advantages of the IBI analysis, considering its temporal resolution^30–32^.

An ECG signal was recorded via the positioning of two electrodes on the right and left intercostal muscles, and a reference electrode on the anterior/inferior side of the right tibia. Both data acquisition and data analysis were performed using BIOPAC® technology with the Acknowledge® software package (Biopac Inc.).

An initial phase of data pre-processing was performed which included: a) 1000 µS/V gain; b) a 0.05 Hz filter (high-pass); and c) a 35 Hz filter (low-pass). After the detection of markers related to the presentation of emotional images from each regulation category, a time recording was obtained for the cardiac interbeat interval (IBI) over the course of the whole experiment. IBI values for 13 separate points were obtained through this procedure (with 1000 ms intervals between each IBI value). The IBI values were categorized as: two points occurring prior to target image presentation (IBI-2 and IBI-1); one point occurring at the exact moment of the start of target image presentation (IBI0); and 10 points occurring after the start of the target image presentation (IBI1 to IBI10). To perform data correction relative to the baseline value, the mean value of IBI-2 (baseline) was subtracted from all the IBI values between IBI-1 and IBI10. As such, the IBI values reported in the results represent the delta values obtained in this procedure.

#### Data Analysis

Statistical analyses were conducted using Statistica software (Stat-Soft Inc., version 8.0). Initially, in order to exclude any difference among groups we performed a One-way ANOVA on each of the pre-test scales. Next, the scores on positive and negative affect were separately analysed using repeated-measures analysis of variance (ANOVA), using the experimental group (tDCS Montage) and Time (before and after the experimental paradigm) as the factors. Regarding the Emotional Experience we performed separate repeated measures ANOVA for each emotional dimension (valence and arousal) with the experimental group (tDCS Montage) and Strategy (Up-regulation, Down-regulation and Maintain) as the factors. Finally, a repeated-measures ANOVA was performed for all IBI values using the experimental group (tDCS Montage), Strategy, and IBI event (IBI-1 to IBI10) as the factors. Where a significant difference was found between the factors, Duncan *post hoc* test was used since multiple comparisons were performed.

### Results

#### Pre-test scales

A one-way ANOVA revealed no statistical differences for any of the scales (see Table 1), except for age.

**Table 1:** Statistical analysis of each scale/questionnaire for each experimental group.

	ANF3/CATF4 (n30)	ANF4/CATF3 (n30)	SHAM (n30)	F	*p*
Age	23,1 (0,6)	22,0 (0,6)	20,8 (0,6)	3.90	0.02*
BAI	4,6 (0,8)	6,1 (0,9)	5,9 (0,9)	0.96	0.39
BDI	5,4 (0,9)	6,8 (1,0)	7,7 (0,9)	1.62	0.20
ERQ - CR	31,2 (1,2)	27,7 (1,3)	29,6 (1,3)	1.83	0.17
ERQ - S	13,7 (0,8)	13,1 (0,9)	15,1 (0,9)	1.38	0.26
Edinburgh	75,9 (3,9)	70,8 (4,2)	73,6 (4,0)	0.40	0.67

The values for each group represent mean and standard error, as well as the F and *p* values. *Significant effect for age difference between experimental groups.

#### Effect of tDCS on Affect

The levels of positive and negative affect were analysed separately. For levels of negative affect, a repeated-measure ANOVA revealed a significant main effect of Time (F_1,87_ = 46.052; *p* < 0.001; η_p_ = 0.346) but not of tDCS Montage (F_2,87_ = 0.342; *p* = 0.711; η_p_ = 0.008), or the interaction between tDCS Montage*Time (F_2,87_ = 1.680; *p* = 0.192; η_p_ = 0.037). With respect to the significant main effect observed for Time, greater levels of negative affect following the experimental task (22.90 ± 0.81) were observed as compared to the levels observed prior to the task (17.17 ± 0.65), revealing a significant increase of negative affect by the task.

With respect to the analysis of levels of positive affect, a repeated-measures ANOVA revealed a significant main effect for Time (F_1,87_ = 83.592; *p* < 0.001; η_p_ = 0.490) but not for tDCS Montage (F_2,87_ = 0.287; *p* = 0.752; η_p_ = 0.007), or the interaction between tDCS Montage*Time (F_2,87_ = 0.412; *p* = 0.663; η_p_ = 0.009). With respect to the significant main effect observed for Time, it was observed lower levels of positive affect following the experimental task (27.04 ± 0.72) as compared to the levels observed prior to the task (33.33 ± 0.59) consistent with the levels of negative affect, revealing a significant decrease of positive affect by the task.

#### Effect of Reappraisal Strategy and tDCS on Emotional Experience

A repeated-measures ANOVA was conducted on the scores obtained from the emotional valence estimation, revealing a significant main effect of Strategy (F_2,174_ = 100.94; *p* < 0.001; η_p_ = 0.537) but not of tDCS Montage (F_2,87_ = 0.015; *p* = 0.985; η_p_ < 0.001), or the interaction between tDCS Montage*Strategy (F_4,174_ = 0.735; *p* = 0.569; η_p_ = 0.017). As a significant main effect was observed for Strategy, Duncan *post hoc* tests were performed, which showed significant differences (p < 0.001) between all three strategies. In particular, the valence estimation scores in the Up-regulation strategy (2.39 ± 0.08) were significantly lower than the scores observed for the Down-regulation strategy (3.50 ± 0.11) and the Maintain strategy (2.82 ± 0.10). In addition, the scores observed for the Down-regulation strategy were significantly higher than the scores observed for the Maintain strategy. In terms of the emotional valence scale, the attribution of a lower score corresponds to the perception of a more negative emotional valence.

**Figure 2:**
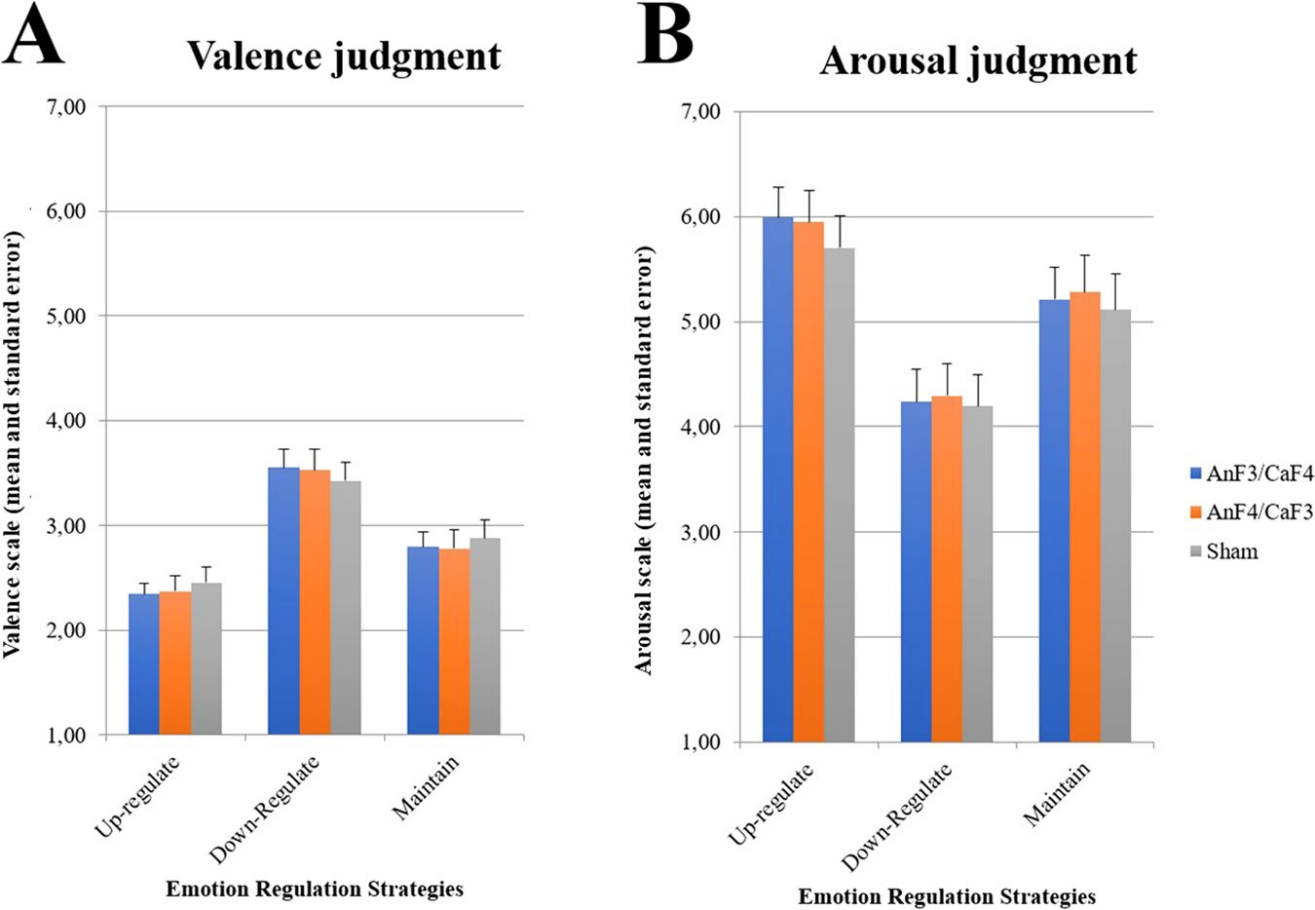
Representation of the emotional experience (**A** – Valence judgment; **B** – Arousal judgment) average scores for negative pictures in respect to the reappraisal strategies and tDCS montages.

With respect to arousal, a repeated-measures ANOVA revealed a significant main effect of Strategy (F_2,174_ = 139.528; *p* < 0.001; η _p_ = 0.616) but not of tDCS Montage (F_2,87_ = 0.526; *p* = 0.593; η_p_ = 0.012), or the interaction between tDCS Montage*Strategy (F_4,174_ = 0.406; *p* = 0.804; η_p_ = 0.009). As a significant main effect was observed for Strategy, Duncan *post hoc* tests were performed, which showed significant differences (p < 0.001) between all three strategies. In particular, the arousal estimation scores in the Up-regulation strategy (5.89 ± 0.17) were significantly higher than the scores observed for the Down-regulation strategy (4.25 ± 0.18) and the Maintain strategy (5.21 ± 0.19). In addition, the scores observed for the Down-regulation strategy were significantly lower than the scores observed for the Maintain strategy. In terms of the emotional arousal scale, the attribution of a higher score corresponds to the perception of greater emotional arousal. Figure 2 shows the graph with the combination of the emotional experience results.

#### Effect of Reappraisal Strategy and tDCS on Interbeat Interval (IBI)

**Figure 3:**
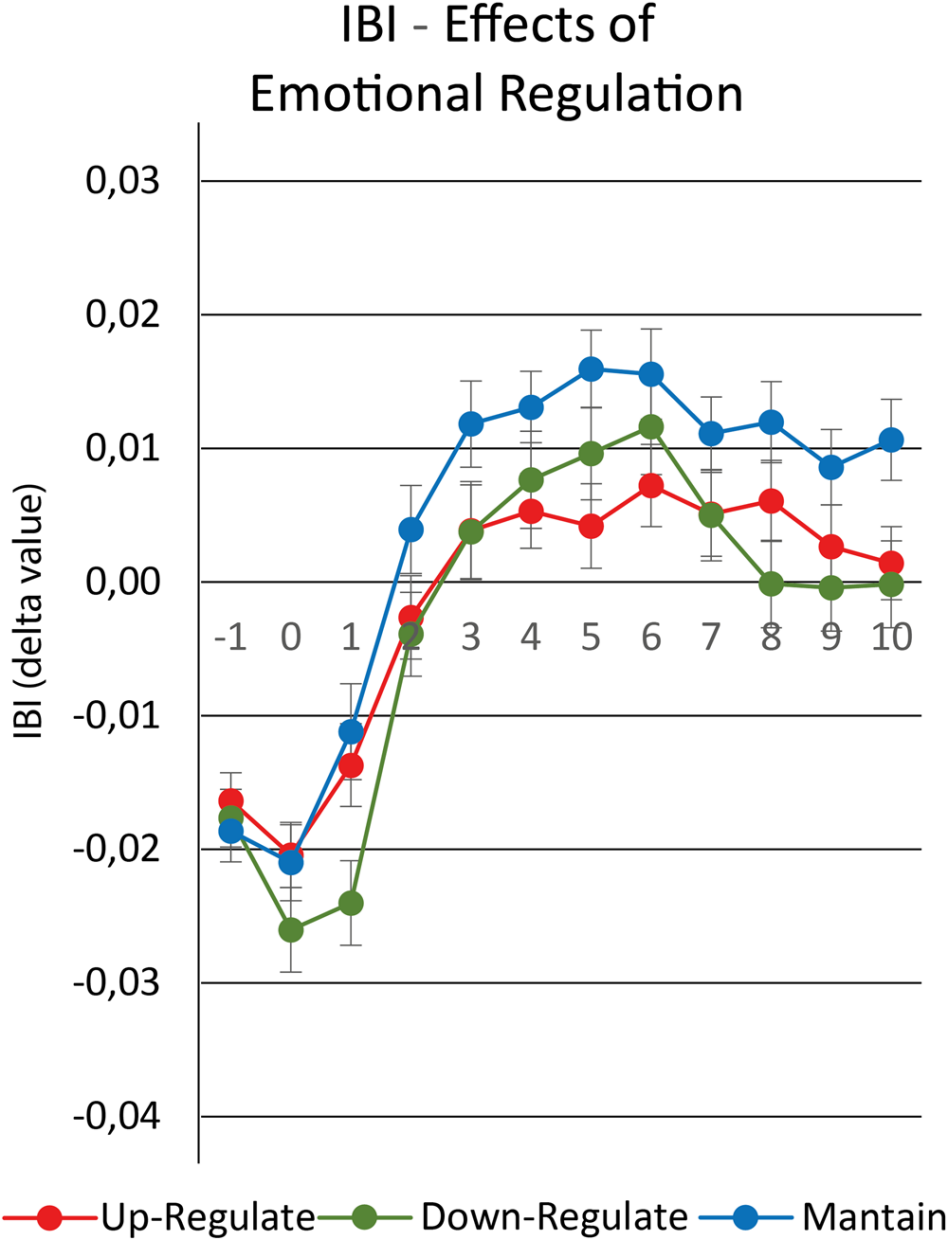
Representation of IBI variation by strategy. Lines are divided by strategy, where values represent IBI delta value,and spreads represent standard error.

A repeated-measures ANOVA revealed a significant main effect for the factors of IBI event (F_11,935_ = 49.768; *p* < 0.001; η_p_ = 0.369), Strategy (F_2,170_ = 4.475; *p* = 0.013; η_p_ = 0.050), and a significant interaction between Strategy*IBI event (F_22,1870_ = 2.687; *p* < 0.001; η_p_ = 0.031) but no significant effects for the factor tDCS Montage (F_2,85_ = 0.700; *p* = 0.500; η_p_ = 0.016) or for the interactions between tDCS Montage*Strategy (F_4,170_ = 0.235; *p* = 0.918; η_p_ = 0.005), tDCS* IBI event (F_22,935_ = 1.097; *p* = 0.343; η_p_ = 0.025), or tDCS*Strategy* IBI event (F_44,1870_ = 1.002; *p* = 0.469; η_p_ = 0.023). Due to the significant effects observed for the factor IBI and the interaction between Strategy* IBI event, Duncan *post hoc* tests were performed, revealing significant differences on all IBI’s, except on IBI-1 (Fig. 3). Specifically to Maintain strategy, it was found significant differences on: IBI0) to Down-regulation (*p* = 0.058); IBI1) to Down-regulation (p < 0.001); IBI2) to Down-regulation (*p* = 0.007) and Up-regulation (*p* = 0.029); IBI3 to Down-regulation (*p* = 0.007) and Up-regulation (*p* = 0.009); IBI4) to Up-regulation (*p* = 0.010); IBI5) to Down-regulation (*p* = 0.035) and Up-regulation (*p* < 0.001); IBI6) to Up-regulation (*p* = 0.005); IBI7) to Down-regulation (*p* = 0.047) and Up-regulation (*p* = 0.049); IBI8) to Down-regulation (*p* < 0.001) and Up-regulation (*p* = 0.052); IBI9) to Down-regulation (*p* = 0.003) and Up-regulation (*p* = 0.062); IBI10) to Down-regulation (*p* < 0.001) and Up-regulation (*p* = 0.002). Finally, in respect to Down-regulation and Up-regulation comparison, it was found significant differences on IBI0 (*p* = 0.040), and IBI1 (*p* < 0.001). No significant differences were found for the other comparisons.

Thus, these IBI’s findings demonstrate that in the first two seconds of emotional processing, the cardiac response is higher on Down-regulation than Up-regulation, and comparing both active regulation to Maintain strategy. Furthermore, regardless of the cognitive reappraisal strategy, the IBI delta value for IBI2 to IBI10 are significant higher for Down-regulation and Up-regulation compared to Maintain strategy (except for IBI4 and IBI6 that only Up-regulation demonstrate significant differences as compared to Maintain strategy).

### Discussion

Experiment 1 sought to investigate the effect of sham tDCS application compared to two experimental tDCS montages with active stimulation over the DLPFC (i. ANF3/CATF4; ii. ANF4/CAF3) during a cognitive reappraisal task of emotional images. The main findings of this Experiment were i. a significant effect of the cognitive reappraisal on the emotional evaluation of the pictures as well as on cardiac interval inter beats and ii. no effect of tDCS on the emotional evaluation.

As previously described by Gross^3^, the current experiment found a significant effect of cognitive control on emotional expression, supporting the increased use of this strategy type in clinical practice^33,34^, but in addition, we found cardiac alterations due to the use of different emotion reappraisal strategies, as first observed by Urry *et al*.^35^.

First of all, the results from ECG are in accordance to several reviews and classic studies in the area^36,37^ that typically after exposure to an emotional stimulus there is an heart rate deceleration. Furthermore, we found that the use of the Down-regulation strategy at the IBI0 and IBI1 events led to a decrease in IBI event when compared to the other strategies. During events between IBI2 to IBI10, lower IBI event were observed for the use of Down- and Up-regulation strategies when compared to the Maintain strategy. In this line, some studies on the cardiac recruitment during cognitive/emotional tasks^30,31^, observed increased IBI event (decrease in cardiac recruitment) after 2000 ms period from the emotional stimulus. These authors considered these findings as a decrease in cognitive engagement after habituation in relation to the exposed emotional stimulus. Here, the finding regarding the use of both active cognitive reappraisal strategies occurs in the opposite direction, that is, a decrease in IBI event (increased cardiac recruitment), which, therefore, following the literature^30–32^, would reflect a greater cognitive engagement. More specifically, in the same way as presented by Urry *et al*.^35^, it can be understood that during the use of both active cognitive reappraisal strategies, the participants should reappraise the cognitive label of the emotional image (diminishing or increasing the negativity of the image) throughout all the presentation time, observed greater cognitive engagement (measured by cardiac recruitment), compared with the Mantain strategy. Thus, our data revealed that there is significant differences in cardiac response when participants employed an active cognitive reappraisal strategy (Up- and Down-regulation) compared to the passive observation (Maintain condition) of images after the IBI2 event. This effect can be understood as the result of the increased cognitive demand during the image presentation time (IBI2 to IBI10) from the two active cognitive reappraisal strategies, where the participants were required to respond appropriately to the emotional image according to an assigned cognitive label, as compared to the passive Maintain strategy. This finding is in accordance with Urry *et al*.^35^, which presented sympathetic activation in respect to the use of active cognitive reappraisal strategies, either by the pupil diameter or by IBI measure.

Additionally, Experiment 1 also revealed significant differences in IBI values between the Down-regulation strategy and the other strategies for the IBI0 and IBI1 events. As IBI0 corresponds to the start of presentation of the emotional image, the lower latencies observed at IBI0 and IBI1 for the Down-regulation strategy may reflect increased cognitive engagement of the subjects in the interval prior to image presentation, at IBI-1 (the instructions screen). This interpretation could be challenged by observing that the same pattern of cardiac response was not found for the Up-regulation strategy. Nevertheless, according to the participants’ self-reports, they had greater difficulty performing the Down-regulation task than the Up-regulation task. Thus, probably this difficulty in decreasing the negative cognitive label of the negative images promoted a greater cognitive engagement immediately after the observation of the instruction screen, an engagement that possibly reflected in higher cardiac recruitment observed at the IBI0 and IBI-1 of the Down-regulation strategy, but not in other strategies. This finding is in accordance with the study by Vanderhasselt *et al*.^38^, which demonstrates that the presentation of an informative screen about the valence of the image to be displayed promotes higher cognitive engagement as measured by the pupil diameter.

Overall however, in contrast to our stated hypotheses, no significant effects of tDCS modulation were found in Experiment 1. Up until the present, only one previous study has aimed to investigate the effects of tDCS applied to the DLPFC during tasks of cognitive reappraisal. Feeser, *et al*.^12^ observed that anodal tDCS application to the right DLPFC, with the cathode/reference electrode placed in the contralateral supraorbital region, led to a potentiation of cognitive control in a task of cognitive reappraisal as compared to a sham tDCS condition. In addition, the authors also found similar effects in terms of the amplitude levels of skin conductance response following the experimental condition of tDCS application. Nevertheless, the results from the current experiment did not replicate these previous findings. It is highly possible that the methodological differences between the study by Feeser *et al*.^12^ and the current experiment, such as the electrode size (35 × 100 cm^2^ vs. 16 × 16 cm^2^, respectively) and the placement of the electrodes (contralateral supraorbital region vs. contralateral homologous region), may have influenced the results observed in the two studies. In addition, the current experiment opted explicitly not to place the reference electrode in the contralateral supraorbital region, as some studies have suggested a role of the medial prefrontal cortex in the employment of strategies of cognitive reappraisal^35,39^, which would be problematic for the interpretation of any results obtained. As such, the current experiment sought to consider the neuromodulatory effects of both the anodal and cathodal pole on the regions over which the electrodes were placed.

Additionally, we modelled our tDCS montage using the toolbox Comets2^26^. As it can be seen in Fig. 1, our bilateral montage (F3/F4) resulted in higher current densities more dorsal than dorsolateral in the PFC which might explain the lack of tDCS effects. Thus, more research are needed to investigate transcranial modulation of the DLPFC in tasks of cognitive reappraisal of emotional stimuli, to gain a greater scientific understanding of the role of this brain are in emotional regulation. New electrode sizes and montages should be modelled and tested in order to enhance the focality of tDCS.

## Experiment 2 – VLPFC tDCS

### Materials and Methods

Experiment 2 used the same stimuli and equipment, and followed the same experimental procedures as Experiment 1, differing only in terms of the tDCS montage as described below. Also, Experiment 2 used the same sample size as Experiment 1 (90 university students) considering all the inclusion/exclusion criteria assumed before. All participants provided written informed consent, and all experiments were performed in accordance with relevant guidelines and regulations.

#### Transcranial Direct Current Stimulation (tDCS)

The equipment used in this experiment were the same as in experiment 1, only differing by the electrodes site positions (based on Riva *et al*.^17^): (1) anode positioned on F7 and cathode positioned on F8 (ANF7/CATF8); (2) anode positioned on F8 and cathode positioned on F7 (ANF8/CATF7); and (3) sham stimulation, with the same montage as condition ANF7/CATF8. Furthermore, as in experiment 1, all participants were blinded for the stimulation condition.

### Results

#### Pre-test scales

Of all 90 participants tested in Experiment 2, only the data from one participant was excluded due a failure in ECG and behavioural data recording. The relative analysis of the demographic questionnaires was conducted on the remaining 89 participants (30 men; mean age of 21.36 ± 3.29 standard error). A one-way ANOVA revealed no statistically significant differences for any of the scales (see Table 2).

**Table 2:** Statistical analysis of each scale/questionnaire for each experimental group. The values for each group represent mean and standard error, as well as the F and *p* values.

	ANF7/CATF8 (n29)	ANF8/CATF7 (n30)	SHAM (n30)	F	*p*
Age	20,62 (0,60)	21,10 (0,59)	22,43 (0,59)	2.50	0.09
BAI	4,86 (0,93)	6,70 (0,92)	7,70 (0,92)	2.40	0.10
BDI	5,83 (0,82)	6,07 (0,81)	8,30 (0,81)	2.85	0.06
ERQ - CR	29,52 (1,36)	29,27 (1,33)	28,97 (1,33)	0.04	0.96
ERQ - S	15,10 (0,96)	13,97 (0,95)	12,70 (0,95)	1.59	0.21
Edinburgh	77,27 (3,69)	80,86 (3,63)	77,11 (3,63)	0.34	0.71

#### Effect of tDCS on Affect

Levels of positive and negative affect were analysed separately. A repeated-measures ANOVA for negative affect revealed a significant main effect for the factor of Time (F_1,86_ = 42.049; *p* < 0.001; η_p_ = 0.328) but not for the factor of tDCS Montage (F_2,86_ = 0.128; *p* = 0.880; η_p_ = 0.003), and with no significant interaction between tDCS Montage*Time (F_2,86_ = 0.410; *p* = 0.665; η_p_ = 0.009). With respect to the significant effect of Time, we observed increased levels of negative affect following the experimental task (22.43 ± 0,80) when compared to the levels observed prior to the task (17.40 ± 0.55), highlighting the role of the task in the modulation of negative affect.

With respect to the analysis of levels of positive affect, a repeated-measures ANOVA revealed a significant main effect for the factor of Time (F_1,86_ = 38.576; *p* < 0.001; η_p_ = 0.310) but not for the factor of tDCS Montage (F_2,86_ = 0.500; *p* = 0.608; η_p_ = 0.012), or the interaction between tDCS Montage*Time (F_2,86_ = 0.636; *p* = 0.532; η_p_ = 0.015). Again, with respect to the significant main effect observed for Time, it was observed lower levels of positive affect after the experimental task (29.25 ± 0.93) when compared to the levels observed prior to the task (33.62 ± 0.71). Similarly to the effect observed for negative affect, this reveals a significant role of the task in the modulation of positive affect as well.

#### Effect of Reappraisal Strategy and tDCS on Emotional Experience

**Figure 4:**
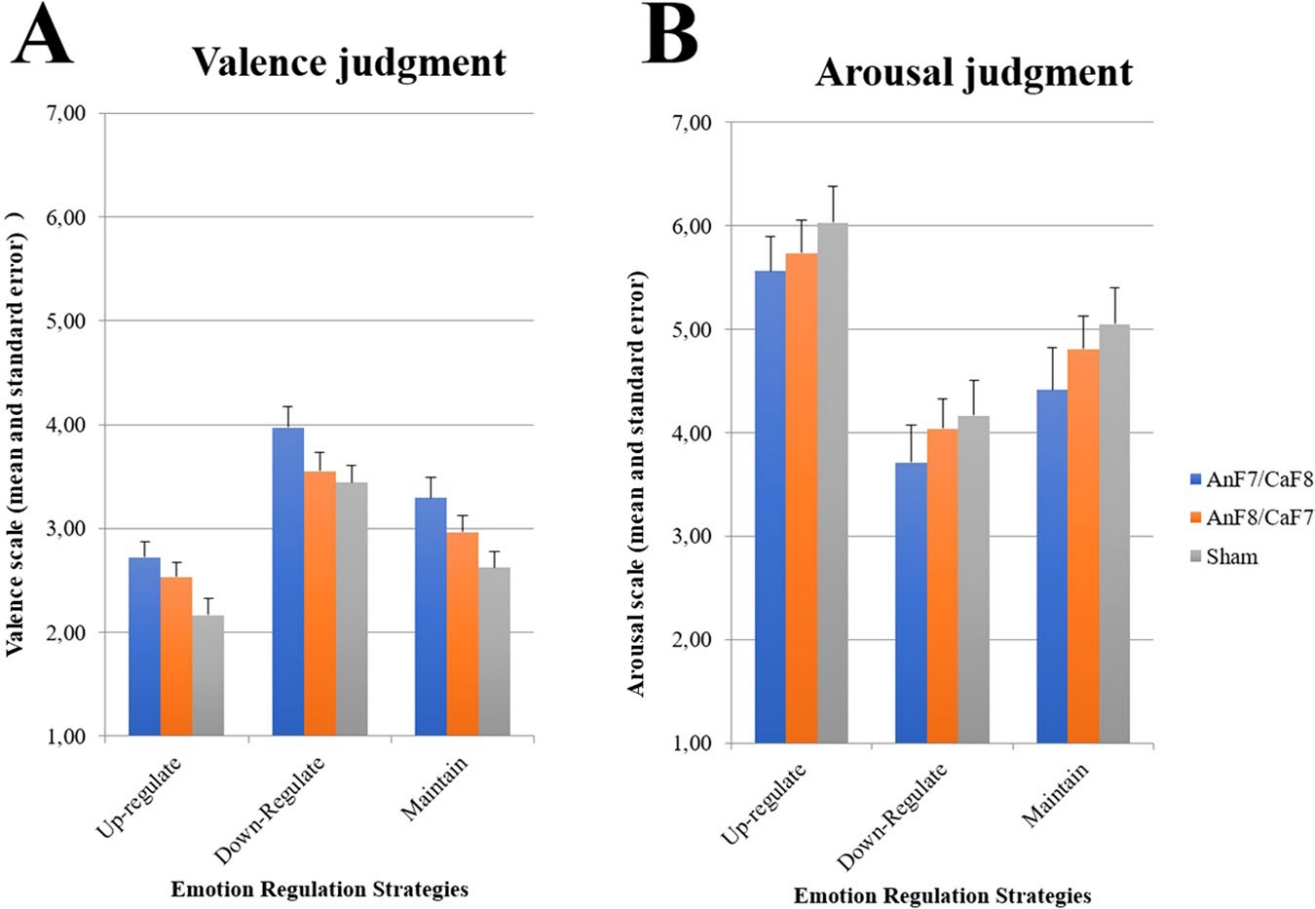
Representation of the emotional experience (**A** – Valence judgment; **B** – Arousal judgment) average scores for negative pictures in respect to the reappraisal strategies and tDCS montages.

A repeated-measures ANOVA revealed a significant main effect for the factors tDCS Montage (F_2,86_ = 3.727; *p* = 0.028; η_p_ < 0.080) and Strategy (F_2,172_ = 135.815; *p* < 0.001; η_p_ = 0.612), but no significant interaction between tDCS Montage*Strategy (F_4,172_ = 0.833; *p* = 0.506; η_p_ = 0.019). As a significant main effect was observed for tDCS Montage, Duncan *post hoc* tests was performed, which showed a significant difference (*p* < 0.010) between the ANF7/CATF8 and the Sham group. That is, regardless of the cognitive reappraisal strategy used, the valence estimation scores observed for the ANF7/CATF8 group (3.33 ± 0.15) were significantly higher than the scores observed for the Sham group (2.74 ± 0.15), but not significantly different from the ANF8/CATF7 group (3.02 ± 0.15), as can be seen in Fig. 4.

Due to the significant effect observed for the factor of Strategy, Duncan *post hoc* tests were performed which revealed significant differences (*p* < 0.001) between all three strategies. Specifically, the valence estimation scores for the Up-regulation strategy (2.47 ± 0.09) were significantly lower than the scores for the Down-regulation strategy (3.66 ± 0.10) and the Maintain strategy (2.96 ± 0.10). In addition, the scores for the Down-regulation strategy were significantly higher than the scores for the Maintain strategy.

With respect to the estimation scores obtained from the arousal rating scale, a repeated-measures ANOVA revealed a significant main effect for the factor of Strategy (F_2,172_ = 124.405; *p* < 0.001; η_p_ = 0.591) but not for the factor of tDCS Montage (F_2,86_ = 0.640; *p* = 0.530; η_p_ = 0.015), or the interaction between tDCS Montage*Strategy (F_4,172_ = 0.252; *p* = 0.908; η_p_ = 0.006). As in Experiment 1, Duncan *post hoc* analysis revealed significant differences (*p* < 0.001) between all three strategies. Specifically, the arousal estimation scores for the Up-regulation strategy (5.78 ± 0.19) were significantly higher than the scores observed for the Down-regulation strategy (3.98 ± 0.19) and the Maintain strategy (4.76 ± 0.21), while the scores for the Down-regulation strategy were significantly lower than the scores for the Maintain strategy.

#### Effect of Reappraisal Strategy and tDCS on Interbeat Interval (IBI)

**Figure 5:**
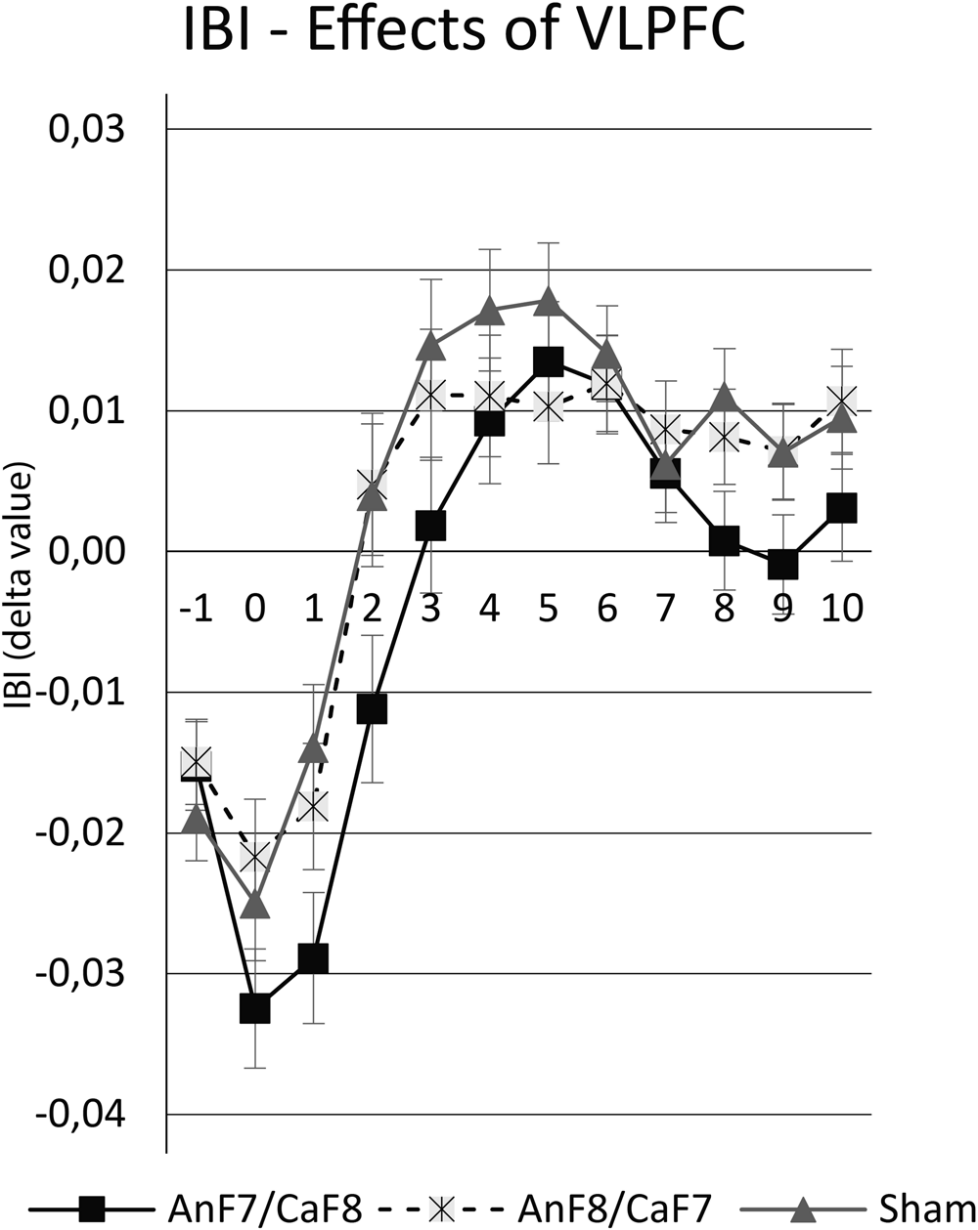
Representation of IBI variation by group. Lines are divided by tDCS group condition, where values represent IBI dela value, and spreads represent standard error.

With respect to the scores obtained for each IBI, a repeated-measures ANOVA revealed a significant main effect for the factor of IBI event (F_11,935_ = 76.407; *p* < 0,001; η_p_ = 0.473) and a significant interaction between tDCS Montage*IBI event (F_22,935_ = 1.690; *p* = 0.025; η_p_ = 0.038), but no significant main effects for the factors of tDCS Montage (F_2,85_ = 1.600; *p* = 0.208; η_p_ = 0.036) Strategy (F_2,170_ = 0.387; *p* = 0.679; η_p_ = 0.005), or the interactions between tDCS Montage*Strategy (F_4,170_ = 1.086; *p* = 0.365; η_p_ = 0.025), Strategy*IBI event (F_22,1870_ = 0.452; *p* = 0.987; η_p_ = 0.005), or tDCS Montage*Strategy*IBI event (F_44,1870_ = 0.955; *p* = 0.556; η_p_ = 0.022). As significant effects were observed for the factor IBI event and the interaction between tDCS Montage*IBI event, Duncan *post hoc* tests were performed, which revealed significant differences between the ANF7/CATF8 group compared to the ANF8/CATF7 (*p* = 0.013) and to the Sham group (*p* = 0.016), specifically for the IBI2 event. Thus, regardless of the cognitive reappraisal strategy used, the IBI2 delta value for the ANF7/CATF8 group (−0.01 ± 0.01) was significantly lower than the values observed for the Sham group (0.00 ± 0.01) and the ANF8/CATF7 group (0.00 ± 0.01), as can be seen in Fig. 5.

### Discussion

Experiment 2 sought to investigate the effect of sham tDCS stimulation as compared to two active tDCS montages (i, ANF7/CATF8; ii, ANF8/CATF7) applied to the DLPFC, during a task of cognitive reappraisal with emotional images.

With respect to the variability of levels of positive and negative affect over the course of the experimental task, the results from Experiment 2 replicated those from Experiment 1. As such, these findings once again confirm the efficacy of the experimental task in the modulation of emotional valence and arousal, and more specifically the levels of affect experienced by the participant. Concurrently, the results obtained from the estimation of valence and arousal were at similar levels in both experiments, revealing the stability of the phenomenon under investigation and confirming the effectiveness of using strategies of cognitive reappraisal as a means of emotional regulation.

Furthermore, in contrast to the results observed in Experiment 1, the current experiment found significant effects of tDCS stimulation on estimation of emotional valence. These findings reveal the specific effect of the ANF7/CATF8 tDCS montage on valence estimation, with a resulting increase in valence estimation scores following stimulation, regardless of the type of cognitive reappraisal strategy employed. Consequently, participants who received ANF7/CATF8 stimulation reported negative emotional images as having less negative valence than participants in the other tDCS conditions, regardless of the cognitive reappraisal strategy employed. This result suggests that ANF7/CATF8 stimulation facilitated a less negative emotional perception of the emotional images.

As Ochsner and Gross^40^ present in their review, ventral regions of Prefrontal Cortex may influence on individual context-appropriate emotional value, while dorsal regions may influence on cognitive control of emotion. In this line, some studies demonstrated the role of VLPFC on mediating Amygdala activity in respect to negative stimuli^41,42^. Our findings on emotional experience judgment and not on specific cognitive reappraisal strategies support this model. tDCS induced effects on the VLPFC led to less negative emotional perception of the emotional images which might be explained to this VLPFC-Amygdala mediation. It is important to note that we manipulated negative images only, which prevent us to evaluate the impact of this modulation on the emotional experience relative to different emotional valence pictures, as well as to evaluate possible differences related to emotional valence. Therefore, we cannot exclude the possibility of a generalized effect for both positive and negative emotional stimuli.

Another point, is the fact that we did not found any significant effect for ANF8/CATF7 as Riva and colleges found in respect to social pain tasks^16,17^. As previously discussed on Experiment 1, these conflicting findings can be understood due to possible methodological differences and experimental purpose. Moreover, a recent meta-analysis of fMRI and emotion regulation studies demonstrate that left Inferior Frontal Gyrus (IFG) and left VLPFC activate during emotion regulation regardless of strategy, but both regions present an important role on selective attention, response inhibition and reorienting attention^42^. Thus, future studies that aim to understand the role of VLPFC tDCS on emotional regulation should separately investigate the effects on cognitive reappraisal and attentional distraction.

Experiment 2 revealed a decrease in IBI values for the IBI0 event during ANF7/CATF8 tDCS stimulation as compared to the Sham condition, and a similar decrease for the IBI1 condition during ANF8/CATF7 stimulation as compared to the Sham condition. Thus, regardless of the type of cognitive reappraisal strategy employed, the participants who received ANF7/CATF8 stimulation showed an increased heart rate in the two seconds immediately preceding the presentation of the experimental instructions. This suggests that the participants showed a greater level of cognitive engagement during ANF7/CATF8 stimulation at the initial instructions screen than participants receiving other tDCS conditions, which may have influenced participants increased valence estimation, compared to the other tDCS conditions. Interestingly, this finding was not limited to the specific employment of one type of cognitive reappraisal strategy but was a generalized effect across all strategies. Thus, we understand that ANF7/CATF8 stimulation may produce an “*physiological compensatory effect*”, in which, in response to extreme negative picture observation, physiological recruitment increases to decrease subjective emotional experience.

Together, these findings represent the causal effect of ANF7/CATF8 stimulation on the emotional experience and physiological modulation of a negative picture observation task. However, it is important to note that the opposite tDCS montage, relative to ANF8/CATF7 stimulation did not significantly influenced the same phenomena. Relative to the classical tDCS mechanisms in respect of polarity dependent effect, it can be considered that both electrodes, anode at F7 and cathode at F8 effectively impacted the target neural population, with the neural population in ANF7 being predominantly excited and the population in CATF8 inhibited. So, the observed effect of ANF7/CATF8 stimulation on emotional experience and physiological modulation is characterized by the sum of individual effects of the positioning of the anode and cathode electrodes. In this sense, the opposite montage should have the opposite result, but this result was not found. One possible explanation could be characterized by the specific lateralized response in relation to each type of tDCS montage.

As presented by Rêgo *et al*.^14^, two contralateral PFC balanced tDCS montage (ANF3/CATF4 and ANF4/CATF3) could present two different effects even though they do not have opposite characteristics. Rêgo *et al*.^14^ demonstrate that during a pain-related video observation task, both ANF3/CATF4 and ANF4/CATF3 tDCS effectively modulate valence/arousal evaluation and pupil dilatation response. Nevertheless, they demonstrate that, considering the results specificity, each tDCS montage present a particular effect, resultant of the combination of the modulation of both electrodes. Thus, in our study, despite the consideration of the opposite effects expected from opposing tDCS montages, the absence of opposing effects can be accepted, considering a sum of other effects and phenomena not controlled, such as: measure the effective cortical excitability modulation, interparticipant variability, and uni-hemispheric controlled stimulation.

### General Conclusion

Overall, the current study sought to investigate the role of prefrontal cortex (PFC), specifically the dorsolateral PFC (Experiment 1) and the ventrolateral PFC (Experiment 2) in different strategies of cognitive reappraisal, considering the effects of inter-hemispheric differences on behavioural and psychophysiological measures.

With respect to the variability of affect levels, the findings from both Experiment 1 and 2 are consistent with the results commonly observed in the literature with negative emotional images exposure. Concurrently, the data from the estimation scales of emotional valence and arousal in both experiments are also consistent with the support for the efficacy of cognitive reappraisal of emotional stimuli described in the literature. In addition, the ECG data expressed in terms of IBI values from Experiment 1 are an innovative measure of the significant effects of the Down-regulation strategy on the cognitive reappraisal of emotional images.

On the other hand, the findings from Experiment 1 depart from previous hypotheses in the literature, as no significant effects of increased cognitive control of emotions were found from the application of anodal tDCS to the right DLPFC (ANF4/CATF3), which necessitates greater discussion of the standardization of tDCS experimental protocols in a research setting, surrounding both electrode size and the set-up parameters between the target and reference electrodes.

Nevertheless, Experiment 2 showed that the excitatory modulation of the left VLPFC and inhibitory modulation of right VLPFC (ANF7/CATF8 condition), but not inverted condition (ANF8/CATF7) resulted in an additional modulation of the emotional impact of negatively valent images. This result highlights the role of the VLPFC in the process of cognitive reappraisal, a finding which, to our knowledge, has been rarely addressed in the literature. However, once we did not find any significant effect for ANF8/CATF7 condition, the role of VLPFC on the cognitive reappraisal should be understood with caution.

As such, more research should be conducted to better describe the inter-hemispheric differences of the prefrontal cortex in the employment of strategies of cognitive reappraisal of emotional stimuli, as well as the functional differences between the substructures located in this cortical region. Thus, our main limitations may be characterized by the unique experimentation of negative emotions rather than both emotional valences. Future studies should investigate the impact of cognitive reappraisal on positive and negative emotions, as well as the impact of DLPFC and VLPFC tDCS stimulation, through unilateral and bilateral balanced montages. Besides that, it would be interesting to investigate the effect of these conditions on the modulation of the eye movement and eye fixation patterns, by adding attentional distraction as a main emotion regulation strategy, thus allowing the hypothesis testing of the causal role of left VLPFC in the attentional direction process regarding selective attention. Furthermore, here we tested two pre-frontal structures that have relative proximity, in this way it could be considered that the neuromodulation of one region could modulate the other, which would highlight the use of a more focal technique. High-definition tDCS has been shown promising as computational models have shown its better focality as compared to the conventional tDCS (the one used in our study). However, when these two approaches were directly compared, no significant effects emerged between them and both were effective in modulating the performance on a behavioral paradigm^43^. Thus, even resulting in a more diffuse pattern of current flow, the behavioral effects of conventional tDCS seem specifically related to the target areas.

Finally, studies with the joint accomplishment of active tDCS and neuroimaging technique may allow the effective evaluation of cortical modulation dependent on the type of tDCS montage, performed together with the possible behavioural and physiological modulation resulted from this intervention. Nevertheless, our study effectively contributes to the emotion regulation topic, presenting results related to the neural and psychophysiological mechanisms behind this important phenomenon.

## Electronic supplementary material

Appendix

## Data Availability

The datasets generated during and/or analyzed during the current study are available from the corresponding author on reasonable request.

## References

[1] Ekman P (1992). An argument for basic emotions. Cognition & emotion.

[2] Ochsner KN (2004). For better or for worse: neural systems supporting the cognitive down-and up-regulation of negative emotion. Neuroimage.

[3] Gross JJ (2015). Emotion regulation: Current status and future prospects. Psychological Inquiry.

[4] Ochsner Kevin N., Silvers Jennifer A., Buhle Jason T. (2012). Functional imaging studies of emotion regulation: a synthetic review and evolving model of the cognitive control of emotion. Annals of the New York Academy of Sciences.

[5] Ochsner KN, Bunge SA, Gross JJ, Gabrieli JD (2002). Rethinking feelings: an FMRI study of the cognitive regulation of emotion. Journal of cognitive neuroscience.

[6] Kalisch R (2009). The functional neuroanatomy of reappraisal: time matters. Neuroscience & Biobehavioral Reviews.

[7] Buhle JT (2014). Cognitive reappraisal of emotion: a meta-analysis of human neuroimaging studies. Cerebral cortex.

[8] Nitsche MA (2008). Transcranial direct current stimulation: state of the art 2008. Brain stimulation.

[9] Brunoni AR (2012). Clinical research with transcranial direct current stimulation (tDCS): challenges and future directions. Brain stimulation.

[10] Boggio, P. S., Rêgo, G. G., Marques, L. M. & Costa, T. L. Social Psychology and Noninvasive Electrical Stimulation. *European Psychologist* (2016).

[11] Nitsche MA (2005). Modulating parameters of excitability during and after transcranial direct current stimulation of the human motor cortex. The Journal of physiology.

[12] Feeser M, Prehn K, Kazzer P, Mungee A, Bajbouj M (2014). Transcranial direct current stimulation enhances cognitive control during emotion regulation. Brain stimulation.

[13] Pripfl J, Lamm C (2015). Focused transcranial direct current stimulation (tDCS) over the dorsolateral prefrontal cortex modulates specific domains of self-regulation. Neuroscience research.

[14] Rêgo GG (2015). Hemispheric dorsolateral prefrontal cortex lateralization in the regulation of empathy for pain. Neuroscience letters.

[15] Lantrip C, Gunning FM, Flashman L, Roth RM, Holtzheimer PE (2017). Effects of transcranial magnetic stimulation on the cognitive control of emotion: potential antidepressant mechanisms. The journal of ECT.

[16] Riva P, Romero Lauro LJ, DeWall CN, Bushman BJ (2012). Buffer the pain away: stimulating the right ventrolateral prefrontal cortex reduces pain following social exclusion. Psychological science.

[17] Riva P, Romero Lauro LJ, DeWall CN, Chester DS, Bushman BJ (2014). Reducing aggressive responses to social exclusion using transcranial direct current stimulation. Social cognitive and affective neuroscience.

[18] Oldfield RC (1971). The assessment and analysis of handedness: the Edinburgh inventory. Neuropsychologia.

[19] Boian A, Soares D, Silva J (2009). Questionário de Regulação Emocional adaptado para a população brasileira. Retrieved December.

[20] Gorenstein C, Andrade L (1996). Validation of a Portuguese version of the Beck Depression Inventory and the State-Trait Anxiety Inventory in Brazilian subjects. Brazilian journal of medical and biological research = Revista brasileira de pesquisas medicas e biologicas.

[21] Siqueira M, Martins M, Moura O (1999). Construção e validação fatorial da EAPN: Escala de Ânimo Positivo e Negativo. Revista da Sociedade de Psicologia do Triângulo Mineiro.

[22] Bradley MM, Lang PJ (1994). Measuring emotion: the self-assessment manikin and the semantic differential. Journal of behavior therapy and experimental psychiatry.

[23] Ribeiro RL, Pompéia S, Bueno OFA (2005). Comparison of brazilian and american norms for the international affective picture system (IAPS). Revista Brasileira de Psiquiatria.

[24] Lang, P. J. International affective picture system (IAPS): Affective ratings of pictures and instruction manual. *Technical report* (2005).

[25] Jasper H (1958). Report of the committee on methods of clinical examination in electroencephalography. Electroencephalogr Clin Neurophysiol.

[26] Lee C, Jung Y-J, Lee SJ, Im C-H (2017). COMETS2: an advanced MATLAB toolbox for the numerical analysis of electric fields generated by transcranial direct current stimulation. Journal of neuroscience methods.

[27] Brunoni AR (2013). Heart rate variability is a trait marker of major depressive disorder: evidence from the sertraline vs. electric current therapy to treat depression clinical study. International Journal of Neuropsychopharmacology.

[28] Brunoni AR (2013). Polarity-and valence-dependent effects of prefrontal transcranial direct current stimulation on heart rate variability and salivary cortisol. Psychoneuroendocrinology.

[29] Rossi S, Santarnecchi E, Valenza G, Ulivelli M (2016). The heart side of brain neuromodulation. Phil. Trans. R. Soc. A.

[30] Gunther Moor B, Bos MG, Crone EA, van der Molen MW (2014). Peer rejection cues induce cardiac slowing after transition into adolescence. Developmental psychology.

[31] Gunther Moor B, Crone EA, van der Molen MW (2010). The heartbrake of social rejection: heart rate deceleration in response to unexpected peer rejection. Psychological Science.

[32] Bradley MM (2009). Natural selective attention: Orienting and emotion. Psychophysiology.

[33] Norton, P. J. & Paulus, D. J. Toward a unified treatment for emotional disorders: update on the science and practice. *Behavior Therapy* (2015). 10.1016/j.beth.2015.07.002 27993337

[34] Webb TL, Miles E, Sheeran P (2012). Dealing with feeling: a meta-analysis of the effectiveness of strategies derived from the process model of emotion regulation. Psychological bulletin.

[35] Urry HL, van Reekum CM, Johnstone T, Davidson RJ (2009). Individual differences in some (but not all) medial prefrontal regions reflect cognitive demand while regulating unpleasant emotion. Neuroimage.

[36] Lacey, J. I. Somatic response patterning and stress: Some revisions of activation theory. *Psychological stress: Issues in research*, 14–42 (1967).

[37] Lacey, J. Some autonomic-central nervous system interrelationships. *Physiological correlates of emotion*, 205–227 (1970).

[38] Vanderhasselt, M.-A., Remue, J., Ng, K. K. & De Raedt, R. The interplay between the anticipation and subsequent online processing of emotional stimuli as measured by pupillary dilatation: the role of cognitive reappraisal. *Frontiers in psychology* **5** (2014). 10.3389/fpsyg.2014.00207 PMC3952078 24659976

[39] Etkin A, Büchel C, Gross JJ (2015). The neural bases of emotion regulation. Nature Reviews. Neuroscience.

[40] Ochsner KN, Gross JJ (2005). The cognitive control of emotion. Trends in cognitive sciences.

[41] Wager TD, Davidson ML, Hughes BL, Lindquist MA, Ochsner KN (2008). Prefrontal-subcortical pathways mediating successful emotion regulation. Neuron.

[42] Morawetz, C., Bode, S., Derntl, B. & Heekeren, H. R. The effect of strategies, goals and stimulus material on the neural mechanisms of emotion regulation: A meta-analysis of fMRI studies. *Neuroscience & Biobehavioral Reviews* (2016). 10.1016/j.neubiorev.2016.11.014 27894828

[43] Hogeveen J (2016). Effects of high-definition and conventional tDCS on response inhibition. Brain stimulation.

