# Supplementary material for: Ventrolateral but not Dorsolateral Prefrontal Cortex tDCS effectively impact emotion reappraisal – effects on Emotional Experience and Interbeat Interval
Source: Sci Rep. 2018 Oct 17;8:15295. doi: 10.1038/s41598-018-33711-5 (PMC6193012; doi:10.1038/s41598-018-33711-5)
Supplement: Supplementary file 1 — Appendix [file 41598_2018_33711_MOESM1_ESM.docx]

**Ventrolateral but not Dorsolateral Prefrontal Cortex tDCS effectively impact emotion reappraisal – effects on Emotional Experience and Interbeat Interval**

Lucas M. Marques; Letícia Y. N. Morello; Paulo S. Boggio^*^

Appendix

The IAPS (IAPS; LANG & BRADLEY, 2008) Negative (valence: M=1,53 SD=0,47; arousal: M=8,31; SD=0,32) and Neutral (valence: M=5,07 SD=0,27; arousal: M=3,88; SD=0,31) pictures used in the experiment were the following: i) up regulation of negative pictures - 1040, 1070, 1201, 1300, 3010, 3053, 3060, 3063, 3064, 3071, 3102, 3350, 3500, 6242, 6350, 6360, 6510, 6540, 6834, 9005, 9252, 9253, 9600, 9910; ii) passive view of negative pictures - 1052, 1200, 2730, 3000, 3015, 3051, 3061, 3062, 3080, 3168, 3266, 3550, 6190, 6213, 6313, 6550, 6560, 6570, 9040, 9050, 9120, 9265, 9410, 9921; iii) down-regulation of negative pictures - 1113, 2710, 2800, 3120, 3140, 3150, 3170, 3261, 3301, 3400, 3530, 5971, 6200, 6212, 6250, 6260, 6300, 6370, 6571, 6821, 6940, 9340, 9400, 9420; iv) passive view of neutral pictures - 1616, 2381, 2485, 2600, 5530, 7002, 7004, 7009, 7025, 7031, 7035, 7050, 7100, 7170, 7175, 7183, 7185, 7187, 7205, 7217, 7224, 7235, 7490, 7705.
